# Defective formation of IgA memory B cells, Th1 and Th17 cells in symptomatic patients with selective IgA deficiency

**DOI:** 10.1002/cti2.1130

**Published:** 2020-04-29

**Authors:** Christina Grosserichter‐Wagener, Alexander Franco‐Gallego, Fatemeh Ahmadi, Marcela Moncada‐Vélez, Virgil ASH Dalm, Jessica Lineth Rojas, Julio César Orrego, Natalia Correa Vargas, Lennart Hammarström, Marco WJ Schreurs, Willem A Dik, P Martin van Hagen, Louis Boon, Jacques JM van Dongen, Mirjam van der Burg, Qiang Pan‐Hammarström, José L Franco, Menno C van Zelm

**Affiliations:** ^1^ Department of Immunology Erasmus MC University Medical Center Rotterdam The Netherlands; ^2^ Grupo de Inmunodeficiencias Primarias Universidad de Antioquia UdeA Medellín Colombia; ^3^ Department of Internal Medicine Erasmus MC University Medical Center Rotterdam The Netherlands; ^4^ Clinical Immunology Department of Laboratory Medicine Karolinska Institutet at Karolinska University Hospital Huddinge Sweden; ^5^ Bioceros B.V. Utrecht The Netherlands; ^6^ Department of Immunohematology and Blood Transfusion Leiden University Medical Center Leiden The Netherlands; ^7^ Laboratory for Immunology Department of Pediatrics Leiden University Medical Center Leiden The Netherlands; ^8^ Department of Immunology and Pathology Central Clinical School Monash University and The Alfred Hospital Melbourne VIC Australia; ^9^ The Jeffrey Modell Diagnostic and Research Center for Primary Immunodeficiencies in Melbourne Melbourne VIC Australia

**Keywords:** B‐cell memory, cytokine concentration, IgA, selective IgA deficiency, Th1 cells, Th17 cells

## Abstract

**Objective:**

Selective IgA deficiency (sIgAD) is the most common primary immunodeficiency in Western countries. Patients can suffer from recurrent infections and autoimmune diseases because of a largely unknown aetiology. To increase insights into the pathophysiology of the disease, we studied memory B and T cells and cytokine concentrations in peripheral blood.

**Methods:**

We analysed 30 sIgAD patients (12 children, 18 adults) through detailed phenotyping of peripheral B‐cell, CD8^+^ T‐cell and CD4^+^ T‐cell subsets, sequence analysis of *IGA* and *IGG* transcripts, *in vitro* B‐cell activation and blood cytokine measurements.

**Results:**

All patients had significantly decreased numbers of T‐cell‐dependent (TD; CD27^+^) and T‐cell‐independent (TI; CD27^−^) IgA memory B cells and increased CD21^low^ B‐cell numbers. IgM^+^IgD^−^ memory B cells were decreased in children and normal in adult patients. *IGA* and *IGG* transcripts contained normal SHM levels. In sIgAD children, *IGA* transcripts more frequently used *IGA2* than controls (58.5% vs. 25.1%), but not in adult patients. B‐cell activation after *in vitro* stimulation was normal. However, adult sIgAD patients exhibited increased blood levels of TGF‐β1, BAFF and APRIL, whereas they had decreased Th1 and Th17 cell numbers.

**Conclusion:**

Impaired IgA memory formation in sIgAD patients is not due to a B‐cell activation defect. Instead, decreased Th1 and Th17 cell numbers and high blood levels of BAFF, APRIL and TGF‐β1 might reflect disturbed regulation of IgA responses *in vivo*.

These insights into B‐cell extrinsic immune defects suggest the need for a broader immunological focus on genomics and functional analyses to unravel the pathogenesis of sIgAD.

## Graphical abstract

Selective IgA deficiency patients are defective in CD27^+^ and CD27^−^ IgA memory formation. Decreased Th1 and Th17 cell numbers and high levels of BAFF, APRIL and TGF‐β1 might reflect disturbed regulation of IgA responses *in vivo*.

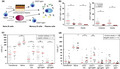

## Introduction

Selective IgA deficiency (sIgAD), the most common primary immunodeficiency in Western countries, is defined by very low to absent serum levels of IgA with normal IgG and IgM.^1^ The prevalence differs between countries and ranges between 1:328 and 1:3040.^2^ The majority of the individuals with absent serum IgA are asymptomatic and identified by coincidental findings.^3^ Still, a number of patients are prone to suffer from recurrent infections and to develop autoimmune diseases and/or allergies.^3^ Altogether, the clinical symptoms of immunodeficiency and immune dysregulation are much higher in sIgAD than in the normal population. This concerns autoimmunity with a prevalence of 25–31% and includes systemic lupus erythematosus, rheumatoid arthritis and type 1 diabetes.^4^, ^5^ In addition, between 18% and 56% of sIgAD patients suffer from one or more allergies.^4^ The pathogenesis of the disease is unknown.

**Figure 1:**
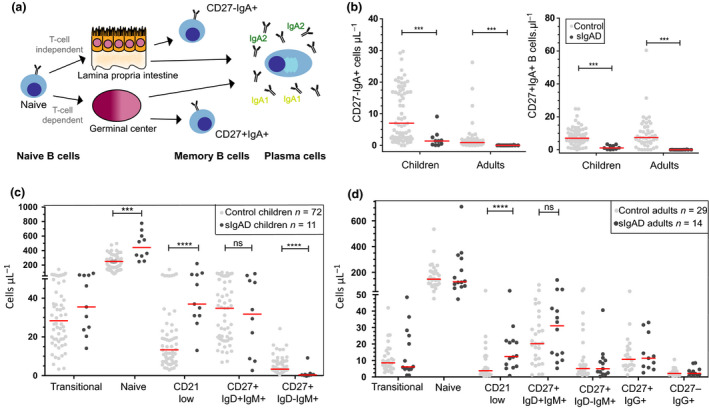
Reductions in CD27^+^ and CD27^−^ IgA^+^ memory B cells in sIgAD patients. **(a)** Schematics of TI and TD IgA responses. **(b)** Quantification of CD27^−^ and CD27^+^ IgA^+^ memory B cells. Adult controls *n* = 29, sIgAD patients *n* = 14; paediatric controls *n* = 67, sIgAD patients *n* = 10. **(c)** Quantification of B‐cell subsets in children and **(d)** adults. The number of patients are indicated in the upper right corner for B‐cell subsets, except for IgG^+^ memory B cells (*n* = 11). **b–d,** red lines indicate median values. Statistics were calculated with the Mann–Whitney *U*‐test; ***P < 0.001, ****P < 0.0001. Technical replicates were not performed. Numbers depict biological replicates.

We and others have shown that IgA memory B cells can derive from T‐cell‐dependent (TD) and T‐cell‐independent (TI) responses (Figure 1a).^6^, ^7^ In human peripheral blood, most IgA memory B cells express CD27 and originate from CD40L‐mediated T‐cell help in germinal centres. In contrast, CD27‐negative IgA memory B cells originate from TI responses in the intestinal tract and are characterised by high IgA2 usage and increased reactivity to intestinal bacteria.^8^ This CD40L‐independent pathway involves the binding of the cytokine A proliferation‐inducing ligand (APRIL) to its receptor transmembrane activator and CAML interactor (TACI), resulting in the expression of activation‐induced cytidine deaminase (AID).^7^, ^9^ Additional cytokines, such as transforming growth factor‐beta (TGF‐β), vasoactive intestinal peptide (VIP) and IL‐10, induce germline *IGA* transcripts leading to *IGA* class switching.^7^, ^10^, ^11^ Previous studies described reduced numbers of Ig class‐switched memory B cells and CD19^+^IgA^+^ B cells in sIgAD patients.^12^, ^13^, ^14^ Recently, Blanco *et al.* grouped sIgAD and other antibody deficiency patients based on the phenotyping of their B‐cell compartment. They proposed that sIgAD patients could segregate into two groups based on differences in IgA^+^ memory B‐cell numbers.^15^ However, the authors did not discriminate between CD27^+^ and CD27^−^ IgA^+^ memory B cells. The analysis of the T‐cell compartment in sIgAD patients has shown that CD4^+^ T cells were reduced.^12^ T‐helper cells in sIgAd have only been studied after *in vitro* stimulation and data on cell counts are lacking.^14^

Several studies have addressed the issue of genetic defects in the pathogenesis of sIgAD. In some patients, chromosome 18p deletions or mutations in *TNFRSF13B*, the gene encoding TACI, have been found.^16^, ^17^, ^18^ However, these genetic variants might be disease‐modifying rather than disease‐causing. Furthermore, distinct HLA haplotypes seem to confer risk for the development of sIgAD.^19^, ^20^ More recently, Bronson *et al.* ^21^ performed a genome‐wide association study (GWAS) meta‐analysis of 1635 patients with IgAD and 4852 controls and found that the *PVT1*, *ATG13–AMBRA1*, *AHI1* and *CLEC16A* genes were significantly associated with sIgAD. Therefore, cytogenetic abnormalities, HLA haplotype associations and known monogenetic disorders are likely involved in the aetiology of sIgAD.^22^

Previously, Wang *et al*.^23^ proposed that the lack of serum IgA results from the absence of *IGA* class switching in B cells in sIgAD patients. They observed that unstimulated peripheral blood mononuclear cells (PBMC) from patients had fewer Cα germline transcripts and Sμ‐Sα fragments than control subjects.^24^, ^25^ However, whether these Cα transcripts are different in somatic hypermutation (SHM) levels or there is preferential class switching towards either *IGA1* or *IGA2* in these patients is currently unknown. It would be noteworthy to solve this in order to gain insight into the potential genetic defects involved in molecular maturation of IgA^+^ B cells.

To study underlying B‐cell intrinsic and extrinsic defects in sIgAD, we performed immunophenotyping of the B‐cell, CD8 T‐cell and CD4 T‐cell compartments, as well as genetic analysis of *IGA* transcripts in children and adults with sIgAD. We also measured cytokine concentrations in blood samples of adult IgAD patients.

Here, we show that both TD‐ and TI‐derived IgA memory B cells are reduced or absent in sIgAD patients. Interestingly, molecular maturation and B‐cell activation were not impaired, but Th1 and Th17 numbers were decreased in adult sIgAD patients. In contrast, we observed increased cytokine concentrations in B‐cell activating factor (BAFF), APRIL and TGF‐β1. Together, these results show that SIgAD patients do not exhibit class switch abnormalities, but a defect in the formation of IgA memory B cells, and Th1 and Th17 cells.

## Results

We included 30 sIgAD patients (12 children, 18 adults) with IgA serum concentrations < 0.07 g L^−1^ (patient details in Supplementary table 1). To obtain more insights into the pathogenesis of sIgAD that might be useful to develop potential treatment strategies, we included mainly symptomatic patients. Twenty‐six out of 30 patients suffered from recurrent respiratory tract infections, 11/25 patients experienced one or more allergies, and 5/27 patients had autoimmune complications. Two adult patients carried heterozygous mutations in *TNFRSF13B*.

### Decreased CD27^−^ and CD27^+^ IgA^+^ memory B cells in sIgAD patients

To investigate whether IgA memory B‐cell formation was affected, we analysed peripheral blood B cells in patients (children *n* = 11, adults *n* = 14) and compared them with healthy controls (children *n* = 67, adults *n* = 29; Figure 1a).^26^, ^27^ We found that both children and adult patients exhibited decreased IgA memory B cells (Figure 1b). Furthermore, IgA memory B cells were undetectable in 11 out of 14 adult patients. In children with sIgAD, we found increased numbers of naive and CD21^low^ B cells, but low IgM^+^IgD^−^ memory B‐cell numbers (Figure 1c). In contrast, in adult patients, only CD21^low^ B cells were increased. IgG memory B cells were studied only in adults, and these were similar to controls (Figure 1d).

Interestingly, some of these abnormalities in peripheral blood B cells of sIgAD patients showed similarities with B‐cell abnormalities observed in CVID patients, such as reduced memory B cells and increased numbers of CD21^low^ B cells.^28^ Still, CVID patients are low in serum IgG, and the different subgroups (as described previously by Driessen *et al.* ^26^) showed additional defects in naive and memory B‐cell subsets that we did not observe in sIgAD patients (Supplementary figure 2). Thus, sIgAD patients showed signs of chronic inflammation as observed by increased CD21^low^ B cells, and these were explicitly defective in IgA B‐cell memory.

### Ig class switching and SHM in transcripts of sIgAD patients

**Figure 2:**
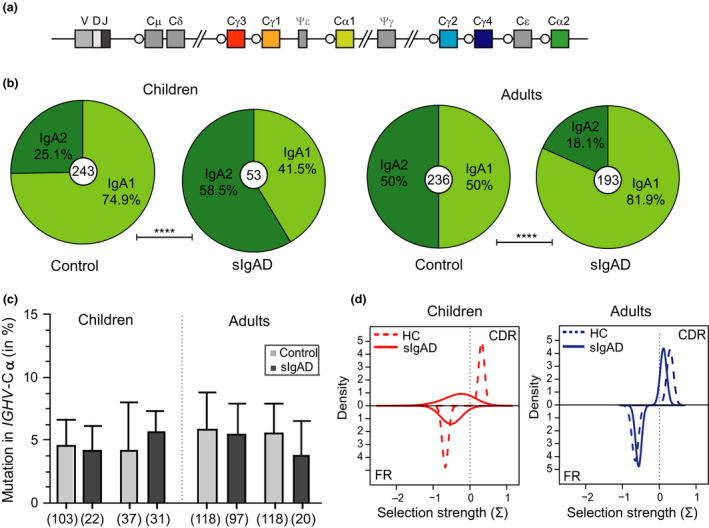
Molecular maturation of *IgA* transcripts in sIgAD patients. **(a)** Schematic overview of the human *IGH* locus depicting the positioning of IgA and IgG encoding regions. **(b)** Distribution of IgA subclasses in children and adults; analysed sequences are indicated with small circles. **(c)** Mutations in *IGA* transcripts (median with interquartile range). Number of sequences analysed shown in parentheses. **(d)** Antigenic selection of *IGA* transcripts of controls (*n* = 477) and sIgAD patients (*n* = 168). **(a–d)** Healthy controls: children *n* = 6, adults *n* = 6. sIgAD patients: children *n* = 6, adults *n* = 9. Statistics were calculated with the Mann–Whitney *U*‐test; ****P < 0.0001. Technical replicates were not performed. Numbers depict biological replicates.

Similar to others,^24^ we detected low numbers of rearranged *IGA* transcripts in PBMCs of patients with sIgAD. We analysed unique sequences to determine Ig subclasses and compared their relative distribution to sequence analysis in controls. *IGA* transcripts from paediatric patients consisted of *IGA2* significantly more often (58.5%) than controls (25.1%) (Figure 2b), whereas in adult patients, *IGA2* transcripts were used less frequently (18.1%) than in controls (50%; Figure 2b). Despite B cells of adult sIgAD patients having reduced usage of *IGA2*, distal *IGG2* subclass usage was normal (Supplementary figure 3a).

Somatic hypermutation (SHM) frequencies in *IGHV* regions in *IGA* transcripts from patients were analysed. We observed that these were similar to controls (Figure 1c). Moreover, we did not observe differences in the selection for replacement mutations in the complementarity determining regions (CDR) (Figure 1d). Likewise, SHM frequencies and selection for replacement mutations in *IGHV* of adult patients' *IGG* transcripts were similar to controls (Supplementary figure 3b and c). Thus, the reduction in *IGA2* usage in sIgAD adults is not due to a general defect in SHM or class switch recombination, but rather a selective defect in IgA responses and memory B‐cell formation.

### Normal B‐cell activation of adult sIgAD patients' naive B cells

**Figure 3:**
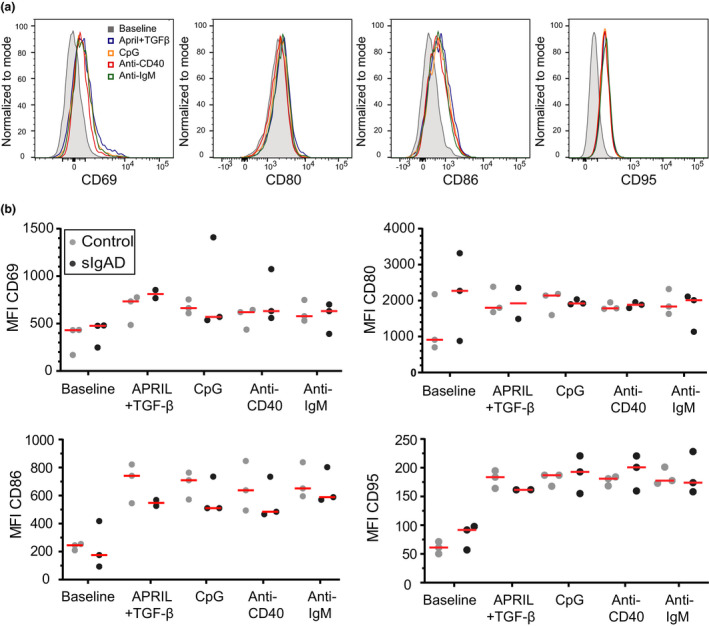
Normal B‐cell activation in adult sIgAD patients. **(a)** Overlays of activation marker expression after 48‐h stimulation of naive B cells from a healthy control. **(b)** MFI (median fluorescent intensity) of activation makers measured 48 h after *in vitro* stimulation of naive B cells from sIgAD adults (*n* = 3; patients 14, 22 and 25) and adult controls (*n* = 3). Technical replicates were not performed. Each experiment was performed on three biological samples per group.

B cells from sIgAD patients have previously been shown to produce IgA following *in vitro* stimulation with CD40L and IL‐21.^29^ However, it is currently unknown whether sIgAD patients show differences in B‐cell activation after short‐term stimulation with TD or TI stimuli. To investigate this, we stimulated peripheral blood B cells from adult patients (patient numbers: 14, 22 and 25) with several TD (anti‐CD40) and TI (anti‐IgM, CpG or APRIL plus TGF‐β) stimuli for 48 h. Cultures with either stimulus resulted in the upregulation of CD80, CD86, CD69 and CD95 on peripheral blood B cells of adult patients and controls (Figure 3). Thus, in symptomatic sIgAD adults, B‐cell activation did not appear to be defective upon stimulation with TD or TI stimuli.

### Reduced Th1 and Th17 cell numbers and increased serum TGF‐β1, APRIL and BAFF levels

**Figure 4:**
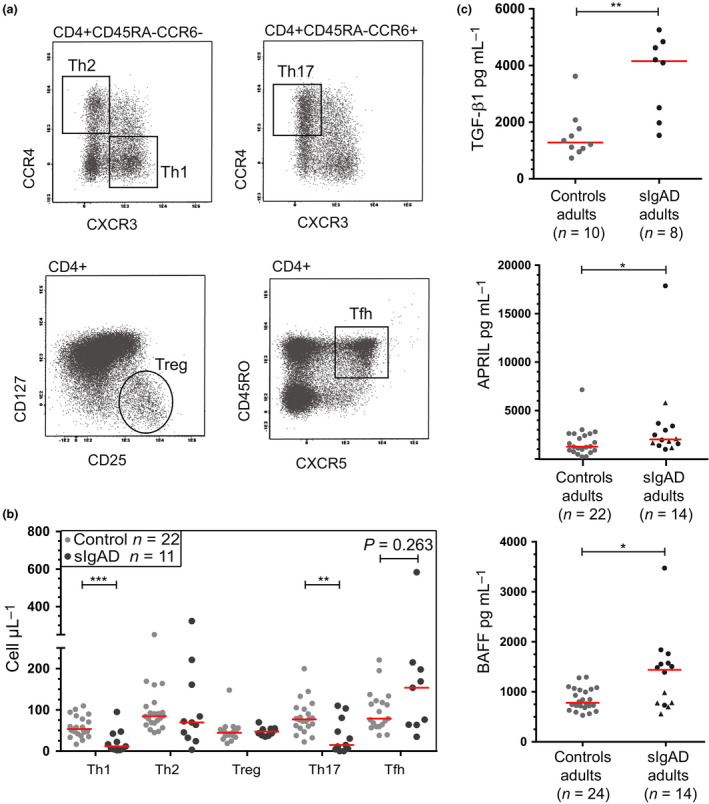
Reduced Th1 and Th17 cell numbers and increased cytokine concentrations. **(a)** Gating strategy of CD4^+^ T cells to define Th1 (CD45RA^−^CCR6^−^CXCR3^+^CCR4^−^), Th2 (CD45RA^−^CCR6^−^CXCR3^−^CCR4^+^), Th17 (CD45RA^−^CCR6^+^CXCR3^−^CCR4^+^), regulatory T cells (Treg; CD25^+^CD127^−^) and follicular helper T cells (Tfh; CD45RO^+^CXCR5^+^). **(b)** Quantification of T‐helper cells in adult sIgAD patients and controls. **(c)** Cytokine concentrations in blood samples (dots represent plasma samples; triangles represent serum samples) of adult sIgAD patients and controls. **(b, c)** Red lines depict median values; statistics were calculated with the Mann–Whitney *U*‐test; *P < 0.05, **P < 0.01, ***P < 0.001. Technical replicates were not performed. Numbers depict biological replicates.

To address a potential B‐cell extrinsic defect in sIgAD, we phenotyped CD4^+^ and CD8^+^ T‐cell subsets and measured peripheral blood concentrations of several cytokines implicated in IgA class switching: TGF‐β1, APRIL and BAFF.^11^, ^30^ We found that IgA‐deficient children had higher numbers of total CD8^+^ T cells, naive CD4^+^ and CD8^+^ T cells, as well as CD4^+^ central memory T cells than controls (Supplementary figure 4a). In sIgAD adults, naive CD4^+^ and CD8^+^, memory CD4^+^, Th2, follicular helper (Tfh) and regulatory (Treg) cell numbers were similar to controls. In contrast, we observed higher numbers of CD8^+^ central memory T cells (Figure 4b) and lower numbers of Th1 and Th17 cells (Figure 4a and b).

Soluble TGF‐β1, BAFF and APRIL were readily detectable in patients' blood and were significantly higher in adult patients than in controls (Figure 4c). Hence, the defect in IgA class switching in our sIgAD patients does not seem to be related to decreased blood concentrations of these cytokines.

## Discussion

In the present study, we searched for underlying B‐cell intrinsic and extrinsic defects in children and adults with sIgAD by characterising the peripheral blood B‐cell and T‐cell compartments, *IGA* transcripts and cytokine concentrations. We observed a reduction in TI‐derived CD27^−^IgA^+^ and TD‐derived CD27^+^IgA^+^ memory B cells in all patients. We did not find an *in vivo* class switch defect to downstream *IGG* and *IGA* subclasses, and affinity maturation of *IGA* transcripts of sIgAD patients was similar to controls. However, patients had reduced Th1 and Th17 cells and increased blood concentrations of TGF‐β1, BAFF and APRIL. Together, these results indicate that sIgAD patients have defective regulation of the IgA response.

Nine out of 12 children and 17 out of 18 adults from our cohort with sIgAD suffered from recurrent infections, and several exhibited atopy and autoimmunity. However, the four asymptomatic patients did not differ immunologically from symptomatic patients. Therefore, symptomatic patients will benefit from a better understanding of the pathophysiology of sIgAD that lead to new treatment strategies. Recently, Blanco *et al.* ^15^ showed that the detection of IgA^+^ memory is not related to the presence of symptoms in sIgAD patients. The majority of adult patients in our cohort had undetectable peripheral IgA^+^ memory B cells, which is higher than in the study of Blanco *et al.*, in which IgA^+^ memory B cells were undetectable in 50% of the patients. The authors defined two IgAD groups: individuals in group 1 had a normal to a mild decrease in number of IgA^+^ memory B cells. In contrast, in group 2, individuals had severe deficiency or undetectable IgA+ memory B cells. The percentages of symptomatic patients did not differ between the two groups. However, sIgAD patients from group 2 experienced more frequently respiratory tract infections and autoimmunity.^15^ Therefore, adult patients in our study may be more comparable to the sIgAD patients of group 2 than group 1.

The generation of IgA2^+^ memory B cells is mainly TI at mucosal sites such as the lamina propria in the intestine.^6^ Our results showing a deficiency of TI‐ and TD‐derived IgA^+^ memory B cells in our patients are in line with the presence of both IgA1^+^ and IgA2^+^ memory B cells in sIgAD patients.^15^

Although by definition, patients with sIgAD have normal IgG, some authors suggest that this disease may be part of a clinical spectrum of disorders such as CVID, based on the observation that some individuals initially diagnosed with sIgAD, progressed over time.^31^, ^32^, ^33^, ^34^ Furthermore, genetic variants in *TNFRSF13B* are more prevalent in both disorders and can coexist within the same family.^16^ However, our two patients in which we found two previously reported *TNFRSF13B* variants associated with CVID did not show additional abnormalities in the B‐cell compartment typical for CVID, such as decreased IgM^+^ or IgG^+^ memory B cells.

Interestingly, the SHM levels and degree of antigenic selection we identified were characteristics of normal affinity maturation of *IGA* and *IGG* in sIgAD patients. In contrast, CVID patients frequently show an abnormal antigen‐selected Ig gene repertoire with reduced SHM levels in *IGA* and *IGG* and abnormalities in subclass distribution such as decreased *IGG2* usage.^35^, ^36^ Our findings of increased *IGA2* usage in children with sIgAD and normal downstream usage of *IGG* subclasses in adults make a class switch defect in sIgAD patients in our cohort unlikely. It remains unclear why *IGA2* transcripts were enriched in children with sIgAD, as this is a relative measure and could result from the overall reduction and a more severe reduction in *IGA1*. Importantly, patients were able to switch to both IgA subclasses *in vivo*, excluding an entire block in Ig switching to one or both *IGA* regions.

Previously, *in vitro* studies had focused on the production of IgA, differentiation into plasmablasts or upregulation of AID.^29^ Our short‐term *in vitro* stimulations showed that TD and TI stimuli did not differ in terms of B‐cell activation.

Patients with predominantly antibody deficiency, including CVID, have reduced numbers of Th17 cells along with increased numbers of CD21^low^ B cells.^36^, ^37^, ^38^ Interestingly, we found decreased Th17 and Th1 cell numbers in adult sIgAD patients. Recently, Lemarquis *et al*.^14^ studied fifteen IgAD patients and did not find abnormalities in Th1 and Th17 cells. These contradictory findings could result because they used the proportion of T helper cells as a fraction of CD4^+^ T cells. Patients with congenital agammaglobulinemia, who lack mature B cells, exhibit decreased frequencies of Th17 cells.^37^ However, these authors questioned the role of B cells in the differentiation of Th17 cells as they showed that in healthy individuals, there is a negative correlation between frequencies of Th17 cells and class‐switched memory B cells and serum concentrations of BAFF.^37^ We observed elevated concentrations of BAFF and APRIL in our adult SIgAD patients. This has also been described for children with sIgAD.^39^ Since total B‐cell numbers were normal in our adult patients, it remains unclear whether the increase in blood BAFF and APRIL levels results from increased production or decreased usage by target cells. In future studies, it would be essential to study dendritic cells as well as T helper cells and cytokine concentrations at local mucosal sites where IgA class switching occurs. Also, it would be interesting to elucidate whether Th17 and Th1 cells are also low in sIgAD patients who have detectable IgA^+^ memory B cells, as well as comparisons of T helper cell numbers between sIgAD patient groups 1 and 2, as recently defined.^15^ These studies might provide further insights into the role of Th17 cells in sIgAD.

Another essential cytokine in the regulation of IgA class switching is TGF‐β1. More than two decades ago, Muller *et al*.,^40^ using a cell bioassay, noted a moderate reduction in serum concentrations of TGF‐β1 in sIgAD patients, and these correlated negatively with the numbers of CD19^+^ B cells.^38^ In contrast, we found increased TGF‐β1 in plasma in five out of 8 patients. Moreover, nearly all patients from our cohort exhibited normal numbers of B cells in peripheral blood. In mice, the lack of TGF‐β receptor expression on B cells leads to defective IgA production.^41^ Therefore, it will be interesting to measure TGF‐β receptor expression, since a significant decrease might explain the high levels of this cytokine observed in some of our patients. Searching for possible genetic defects affecting the signalling cascade of costimulatory molecules beyond *TNFRSF13B* or costimulatory T‐helper cells might provide additional insights into the pathogenesis of sIgAD.

In summary, we show here that in addition to the absence of IgA in blood, symptomatic sIgAD patients are defective in the formation of IgA memory B cells, despite normal B‐cell activation and affinity maturation of their *IGA* and *IGG* transcripts. Instead, decreased Th1 and Th17 cell numbers and high levels of BAFF, APRIL and TGF‐β1 might reflect disturbed regulation of IgA responses *in vivo*. We propose that the severe impairment in IgA class switch in symptomatic sIgAD patients might result from shifts in Th cell subsets and cytokine dysregulation.

## Methods

### Human subjects

We collected clinical data and blood samples of 30 patients (children *n* = 12; adults *n* = 18) with sIgAD and 108 healthy controls (children *n* = 72; adults *n* = 29) after written informed consent (Supplementary table 1). All patients were diagnosed based on the absence of serum IgA (< 0.07 g L^−1^) with normal IgM and IgG. This study was performed according to the Declaration of Helsinki and the guidelines of the Medical Ethics Committees of the Erasmus MC, Karolinska University Hospital, and ethics committee at the University of Antioquia.

### Flow cytometry and cell sorting

Absolute counts of blood CD3^+^ T cells, CD16^+^/56^+^ natural killer cells and CD19^+^ B cells were obtained with a diagnostic lyse‐no‐wash protocol (BD Biosciences, San Jose, CA, USA). Detailed immunophenotyping of B and T cells was performed by 11‐colour flow cytometry using an LSRII Fortessa (BD Biosciences) with a standardised configuration, according to Euroflow protocols.^42^ Data were analysed using FacsDIVA software v8 (BD Biosciences) and Infinicyt software (Cytognos, Salamanca, Spain). Control subjects were described previously.^26^, ^27^ All antibodies used for flow cytometry are listed in Supplementary table 2. The gating strategy of B‐cell subsets is shown in Supplementary figure 1. T‐cell subsets were defined as follows: naive T cells (CD45RO^−^CCR7^+^), central memory (CM) T cells (CD45RO^+^CCR7^+^), effector memory (EM) T cells (CD45RO^+/−^CCR7^−^), Th1 (CD45RA^−^CCR6^−^CXCR3^+^CCR4^−^), Th2 (CD45RA^−^CCR6^−^CXCR3^−^CCR4^+^), Th17 (CD45RA^−^CCR6^+^CXCR3^−^CCR4^+^), regulatory T cells (Treg; CD25^+^CD127^−^) and follicular helper T cells (Tfh; CD45RO^+^CXCR5^+^).^27^, ^43^ Naive mature B cells (CD3^−^CD20^+^CD38^low^CD27^−^IgD^+^) were high‐speed cell sorted to > 95% purity using a FACSAria III (BD Biosciences).

### *In vitro* B‐cell activation and cell cultures

Purified naive mature B cells were cultured at a density of 30 000 cells/200 μL per well in round‐bottom 96‐well plates with RPMI medium (Lonza, Basel, Switzerland) containing 10% foetal calf serum (Thermo Fisher Scientific, Waltham, MA, USA), 1% penicillin/ampicillin and 0.5 μm 2‐mercaptoethanol (Thermo Fisher Scientific). Cells were stimulated for 48 h with either 10 μg mL^−1^ of anti‐CD40 (Bioceros B.V., Utrecht, the Netherlands), 10 μg mL^−1^ F(ab′)2 anti‐IgM (Southern Biotech, Birmingham, AL, USA), 0.5 μm CpG ODN2006 (Invivogen, San Diego, CA, USA) or 1 µg mL^−1^ APRIL (AdipoGen Life Sciences, San Diego, CA, USA) in combination with 0.5 ng mL^−1^ TGF‐β (R&D Systems, Minneapolis, MN, USA) and harvested to determine the expression levels of surface activation marker by flow cytometry (Supplementary table 2).

### Molecular analysis of somatic hypermutations and Ig subclass usage

RNA was isolated from PBMCs with the GeneElute Mammalian Total RNA Miniprep kit (Sigma‐Aldrich, St. Louis, MO, USA), followed by cDNA synthesis. *IGA* and *IGG* transcripts were amplified using *IGHV3* and *IGHV4* leader or FR1 primers in combination with a Cα or Cγ reverse primer.^8^, ^44^ PCR products were cloned into the pGEM‐T easy vector (Promega, Leiden, the Netherlands) and prepared for sequencing on an ABI PRISM 3130XL (Applied Biosystems, Foster City, CA, USA). The sequences obtained were compared with reference sequences from the IMGT database (http://imgt.org). Targeting of SHM in framework regions (FR) and complementarity determining regions (CDR) was analysed with the extended version of IGGalaxy (http://bioinf‐galaxian.erasmusmc.nl/galaxy).^45^ The selection strength for replacement mutations in the FR and CDR was determined with the Bayesian estimation of Antigen‐driven SELectIoN program (http://selection.med.yale.edu/baseline/)^46^, ^47^ IgG and IgA subclasses were determined using the germline sequence of *IGH* locus (NG_001019).

### *TNFRSF13B* mutation analysis

Five exons of the *TNFRSF13B* gene encoding TACI were amplified by PCR (primers listed in Supplementary table 3) and sequenced on an ABI PRISM 3130XL (Applied Biosystems).

### Anti‐IgA antibody analysis

Plasma antibodies directed against IgA (anti‐IgA) were analysed at Sanquin laboratories (Amsterdam, the Netherlands), using the commercially available EliA™ Anti‐IgA enzyme immune assay (Phadia, Thermo Fisher Scientific, Uppsala, Sweden). The assay was performed on the Phadia250 analyser according to the manufacturer's instructions without modification. Reference values used in this assay were < 3 U mL^−1^ negative; 3–10 U mL^−1^ borderline; > 10 U mL^−1^ positive.

### Quantification of BAFF, APRIL and TGF‐β1

An ELISA was used to measure BAFF, APRIL and TGF‐β1 concentrations in plasma and serum samples of sIgAD patients (adults *n* = 14; children *n* = 12) and controls (only adults *n* = 24), according to the manufacturer's instructions (BAFF and TGF‐β1 R&D systems; APRIL, eBioscience, San Diego, CA, USA).

### Statistics

Statistical analyses were performed with the Mann–Whitney *U*‐test or chi‐square test, as indicated in the figure legends. *P*‐values of < 0.05 were considered to be statistically significant as follows: * < 0.05, ** < 0.01, *** < 0.001, **** < 0.0001.

## Conflict of interest

The authors declare no conflict of interest.

## Supporting information

Supplementary Material

## References

[1] Picard C , Bobby Gaspar H , Al‐Herz W *et al* International Union of Immunological Societies: 2017 Primary Immunodeficiency Diseases Committee Report on Inborn Errors of Immunity. J Clin Immunol 2018; 38: 96–128. 29226302 10.1007/s10875-017-0464-9 PMC5742601

[2] Clark JA , Callicoat PA , Brenner NA , Bradley CA , Smith DM Jr . Selective IgA deficiency in blood donors. Am J Clin Pathol 1983; 80: 210–213. 6410901 10.1093/ajcp/80.2.210

[3] Yel L . Selective IgA deficiency. J Clin Immunol 2010; 30: 10–16. 20101521 10.1007/s10875-009-9357-x PMC2821513

[4] Odineal DD , Gershwin ME . The epidemiology and clinical manifestations of autoimmunity in selective IgA deficiency. Clin Rev Allergy Immunol 2020; 58: 107–133. 31267472 10.1007/s12016-019-08756-7

[5] Wang N , Shen N , Vyse TJ *et al* Selective IgA deficiency in autoimmune diseases. Mol Med 2011; 17: 1383–1396. 21826374 10.2119/molmed.2011.00195 PMC3321806

[6] Berkowska MA , Driessen GJ , Bikos V *et al* Human memory B cells originate from three distinct germinal center‐dependent and ‐independent maturation pathways. Blood 2011; 118: 2150–2158. 21690558 10.1182/blood-2011-04-345579 PMC3342861

[7] He B , Xu W , Santini PA *et al* Intestinal bacteria trigger T cell‐independent immunoglobulin A(2) class switching by inducing epithelial‐cell secretion of the cytokine APRIL. Immunity 2007; 26: 812–826. 17570691 10.1016/j.immuni.2007.04.014

[8] Berkowska MA , Schickel JN , Grosserichter‐Wagener C *et al* Circulating human CD27‐IgA+ memory B cells recognize bacteria with polyreactive Igs. J Immunol 2015; 195: 1417–1426. 26150533 10.4049/jimmunol.1402708 PMC4595932

[9] Litinskiy MB , Nardelli B , Hilbert DM *et al* DCs induce CD40‐independent immunoglobulin class switching through BLyS and APRIL. Nat Immunol 2002; 3: 822–829. 12154359 10.1038/ni829 PMC4621779

[10] Fujieda S , Waschek JA , Zhang K , Saxon A . Vasoactive intestinal peptide induces Sα/Sµ switch circular DNA in human B cells. J Clin Invest 1996; 98: 1527–1532. 8833899 10.1172/JCI118944 PMC507583

[11] Zan H , Cerutti A , Dramitinos P , Schaffer A , Casali P . CD40 engagement triggers switching to IgA1 and IgA2 in human B cells through induction of endogenous TGF‐β: evidence for TGF‐β but not IL‐10‐dependent direct Sµ–>Sα and sequential Sµ–>Sγ, Sγ–>Sα DNA recombination. J Immunol 1998; 161: 5217–5225. 9820493 PMC4631047

[12] Nechvatalova J , Pikulova Z , Stikarovska D , Pesak S , Vlkova M , Litzman J . B‐lymphocyte subpopulations in patients with selective IgA deficiency. J Clin Immunol 2012; 32: 441–448. 22328142 10.1007/s10875-012-9655-6

[13] Aghamohammadi A , Abolhassani H , Biglari M *et al* Analysis of switched memory B cells in patients with IgA deficiency. Int Arch Allergy Immunol 2011; 156: 462–468. 21832837 10.1159/000323903

[14] Lemarquis AL , Einarsdottir HK , Kristjansdottir RN , Jonsdottir I , Ludviksson BR . Transitional B cells and TLR9 responses are defective in selective IgA deficiency. Front Immunol 2018; 9: 909–919. 29755476 10.3389/fimmu.2018.00909 PMC5934527

[15] Blanco E , Perez‐Andres M , Arriba‐Mendez S *et al* Defects in memory B‐cell and plasma cell subsets expressing different immunoglobulin‐subclasses in patients with CVID and immunoglobulin subclass deficiencies. J Allergy Clin Immunol 2019; 144: 809–824. 30826363 10.1016/j.jaci.2019.02.017

[16] Castigli E , Wilson SA , Garibyan L *et al* TACI is mutant in common variable immunodeficiency and IgA deficiency. Nat Genet 2005; 37: 829–834. 16007086 10.1038/ng1601

[17] Pan‐Hammarstrom Q , Salzer U , Du L *et al* Reexamining the role of TACI coding variants in common variable immunodeficiency and selective IgA deficiency. Nat Genet 2007; 39: 429–430. 17392797 10.1038/ng0407-429 PMC2931279

[18] Ogata K , Iinuma K , Kammura K , Morinaga R , Kato J . A case report of a presumptive +i(18p) associated with serum IgA deficiency. Clin Genet 1977; 11: 184–188. 837568 10.1111/j.1399-0004.1977.tb01297.x

[19] Olerup O , Smith CI , Hammarstrom L . Different amino acids at position 57 of the HLA‐DQ beta chain associated with susceptibility and resistance to IgA deficiency. Nature 1990; 347: 289–290. 1976229 10.1038/347289a0

[20] Ferreira RC , Pan‐Hammarstrom Q , Graham RR *et al* High‐density SNP mapping of the HLA region identifies multiple independent susceptibility loci associated with selective IgA deficiency. PLoS Genet 2012; 8: e1002476. 22291608 10.1371/journal.pgen.1002476 PMC3266887

[21] Bronson PG , Chang D , Bhangale T *et al* Common variants at PVT1, ATG13‐AMBRA1, AHI1 and CLEC16A are associated with selective IgA deficiency. Nat Genet 2016; 48: 1425–1429. 27723758 10.1038/ng.3675 PMC5086090

[22] Abolhassani H , Aghamohammadi A , Hammarstrom L . Monogenic mutations associated with IgA deficiency. Expert Rev Clin Immunol 2016; 12: 1321–1335. 27266541 10.1080/1744666X.2016.1198696

[23] Wang Z , Yunis D , Irigoyen M *et al* Discordance between IgA switching at the DNA level and IgA expression at the mRNA level in IgA‐deficient patients. Clin Immunol 1999; 91: 263–270. 10370371 10.1006/clim.1999.4702

[24] Asano T , Kaneko H , Terada T *et al* Molecular analysis of B‐cell differentiation in selective or partial IgA deficiency. Clin Exp Immunol 2004; 136: 284–290. 15086392 10.1111/j.1365-2249.2004.02440.x PMC1809032

[25] Islam KB , Baskin B , Nilsson L , Hammarstrom L , Sideras P , Smith CI . Molecular analysis of IgA deficiency. Evidence for impaired switching to IgA. J Immunol 1994; 152: 1442–1452. 8301144

[26] Driessen GJ , van Zelm MC , van Hagen PM *et al* B‐cell replication history and somatic hypermutation status identify distinct pathophysiologic backgrounds in common variable immunodeficiency. Blood 2011; 118: 6814–6823. 22042693 10.1182/blood-2011-06-361881

[27] Heeringa JJ , Karim AF , van Laar JAM *et al* Expansion of blood IgG4^+^ B, TH2, and regulatory T cells in patients with IgG4‐related disease. J Allergy Clin Immunol 2018; 141: 1831–1843.e10. 28830675 10.1016/j.jaci.2017.07.024

[28] Warnatz K , Wehr C , Drager R *et al* Expansion of CD19^hi^CD21l^o/neg^ B cells in common variable immunodeficiency (CVID) patients with autoimmune cytopenia. Immunobiology 2002; 206: 502–513. 12607725 10.1078/0171-2985-00198

[29] Borte S , Pan‐Hammarstrom Q , Liu C *et al* Interleukin‐21 restores immunoglobulin production *ex vivo* in patients with common variable immunodeficiency and selective IgA deficiency. Blood 2009; 114: 4089–4098. 19738033 10.1182/blood-2009-02-207423

[30] Cerutti A , Rescigno M . The biology of intestinal immunoglobulin A responses. Immunity 2008; 28: 740–750. 18549797 10.1016/j.immuni.2008.05.001 PMC3057455

[31] Hammarstrom L , Vorechovsky I , Webster D . Selective IgA deficiency (SIgAD) and common variable immunodeficiency (CVID). Clin Exp Immunol 2000; 120: 225–231. 10792368 10.1046/j.1365-2249.2000.01131.x PMC1905641

[32] Espanol T , Catala M , Hernandez M , Caragol I , Bertran JM . Development of a common variable immunodeficiency in IgA‐deficient patients. Clin Immunol Immunopathol 1996; 80: 333–335. 8811056 10.1006/clin.1996.0132

[33] Carvalho Neves Forte W , Ferreira De Carvalho Junior F , Damaceno N , Vidal Perez F , Gonzales Lopes C , Mastroti RA . Evolution of IgA deficiency to IgG subclass deficiency and common variable immunodeficiency. Allergol Immunopathol (Madr) 2000; 28: 18–20. 10757854

[34] Bonilla FA , Khan DA , Ballas ZK *et al* Practice parameter for the diagnosis and management of primary immunodeficiency. J Allergy Clin Immunol 2015; 136: 1186–2050.e1‐78. 26371839 10.1016/j.jaci.2015.04.049

[35] van Schouwenburg PA , IJspeert H , Pico‐Knijnenburg I *et al* Identification of CVID patients with defects in immune repertoire formation or specification. Front Immunol 2018; 9: 2545. 30532750 10.3389/fimmu.2018.02545 PMC6265514

[36] van Zelm MC , Bartol SJ , Driessen GJ *et al* Human CD19 and CD40L deficiencies impair antibody selection and differentially affect somatic hypermutation. J Allergy Clin Immunol 2014; 134: 135–144. 24418477 10.1016/j.jaci.2013.11.015

[37] Barbosa RR , Silva SP , Silva SL *et al* Primary B‐cell deficiencies reveal a link between human IL‐17‐producing CD4 T‐cell homeostasis and B‐cell differentiation. PLoS One 2011; 6: e22848. 21826211 10.1371/journal.pone.0022848 PMC3149619

[38] Edwards ESJ , Bosco JJ , Aui PM *et al* Predominantly antibody‐deficient patients with non‐infectious complications have reduced naive B, Treg, Th17, and Tfh17 cells. Front Immunol 2019; 10: 2593. 31803177 10.3389/fimmu.2019.02593 PMC6873234

[39] Jin R , Kaneko H , Suzuki H *et al* Age‐related changes in BAFF and APRIL profiles and upregulation of BAFF and APRIL expression in patients with primary antibody deficiency. Int J Mol Med 2008; 21: 233–238. 18204790

[40] Muller F , Aukrust P , Nilssen DE , Froland SS . Reduced serum level of transforming growth factor‐beta in patients with IgA deficiency. Clin Immunol Immunopathol 1995; 76: 203–208. 7614739 10.1006/clin.1995.1116

[41] Borsutzky S , Cazac BB , Roes J , Guzman CA . TGF‐β receptor signaling is critical for mucosal IgA responses. J Immunol 2004; 173: 3305–3309. 15322193 10.4049/jimmunol.173.5.3305

[42] Kalina T , Flores‐Montero J , van der Velden VH *et al* EuroFlow standardization of flow cytometer instrument settings and immunophenotyping protocols. Leukemia 2012; 26: 1986–2010. 22948490 10.1038/leu.2012.122 PMC3437409

[43] Grosserichter‐Wagener C , Radjabzadeh D , van der Weide H *et al* Differences in systemic IgA reactivity and circulating Th subsets in healthy volunteers with specific microbiota enterotypes. Front Immunol 2019; 10: 341. 30899257 10.3389/fimmu.2019.00341 PMC6417458

[44] Tiller T , Meffre E , Yurasov S , Tsuiji M , Nussenzweig MC , Wardemann H . Efficient generation of monoclonal antibodies from single human B cells by single cell RT‐PCR and expression vector cloning. J Immunol Methods 2008; 329: 112–124. 17996249 10.1016/j.jim.2007.09.017 PMC2243222

[45] Moorhouse MJ , van Zessen D , IJspeert H *et al* ImmunoGlobulin galaxy (IGGalaxy) for simple determination and quantitation of immunoglobulin heavy chain rearrangements from NGS. BMC Immunol 2014; 15: 59. 25495099 10.1186/s12865-014-0059-7 PMC4282729

[46] Uduman M , Yaari G , Hershberg U , Stern JA , Shlomchik MJ , Kleinstein SH . Detecting selection in immunoglobulin sequences. Nucleic Acids Res 2011; 39: W499–W504. 21665923 10.1093/nar/gkr413 PMC3125793

[47] Yaari G , Uduman M , Kleinstein SH . Quantifying selection in high‐throughput Immunoglobulin sequencing data sets. Nucleic Acids Res 2012; 40: e134. 22641856 10.1093/nar/gks457 PMC3458526

