# Supplementary material for: Defective formation of IgA memory B cells, Th1 and Th17 cells in symptomatic patients with selective IgA deficiency
Source: Clin Transl Immunology. 2020 Apr 29;9(5):e1130. doi: 10.1002/cti2.1130 (PMC7190975; doi:10.1002/cti2.1130)
Supplement: Supplementary file 1 — Supplementary Material [file CTI2-9-e1130-s001.pdf]

SUPPLEMENTARY TABLES (N=3); FIGURES (N=4)

Supplementary Table 1. Patient characteristics

patient	age (yr)	sex	IgG (g/l)	IgA (g/l)	IgM (g/l)	B cells / µl	NK cells / µl	T cells / µl	$CD4^+$ T cells/ µl	$CD8^+$ T cells/ µl	anti-IgA	TACI variant	recurrent infections	allergies	auto-immune disease	Normal vaccination response	
																Pneumovax	HiB
1	5	M	11.2	**0.04**	0.70	888	520	2806	1269	1121	n.t.	-	+	+	-	n.t.	yes
2	6	F	**14.7**	**<0.04**	1.12	359	116	2112	1247	630	n.t.	-	+	+	-	n.t.	n.t.
3	6	F	**15.0**	**0.00**	1.43	260	276	2810	1586	1016	n.t.	-	+	-	-	n.t.	n.t.
4	6	F	13.3	**0.03**	1.10	877	263	3648	1991	1449	n.t.	-	-	-	-	n.t.	n.t.
5	7	F	11.2	**0.05**	0.56	628	339	3255	1919	916	n.t.	-	+	-	-	n.t.	n.t.
6	9	M	10.5	**0.00**	0.41	423	638	2173	745	1364	n.t.	-	+	+	-	yes	n.t.
7	10	F	**22.1**	**<0.04**	1.20	364	231	3553	1792	1498	n.t.	-	-	+	-	yes	yes
8	10	M	10.5	**0.03**	0.97	498	334	2523	1191	1170	n.t.	-	+	+	-	n.t.	n.t.
9	11	M	11.0	**0.05**	1.26	497	380	1700	909	632	n.t.	-	+	+	-	n.t.	n.t.
10	12	F	**16.7**	**0.04**	0.81	483	269	1843	995	626	n.t.	-	-	+	-	yes	n.t.
11	14	F	10.3	**0.09**	1.22	**718**	200	1547	874	537	n.t.	-	+	-	-	yes	n.t.
12	17	F	12.9	**0.05**	1.17	171	151	1104	555	393	n.t.	-	+	-	SLE	yes	no
13	19	M	16.1	**0.05**	0.70	**1009**	246	1781	990	656	n.t.	-	+	+	-	n.t.	n.t.
14	19	F	16.7	**0.06**	0.36	376	282	1874	1236	536	-	-	+	-	-	yes	n.t.
15	20	M	16.3	**<0.05**	2.09	203	163	873	473	304	-	-	+	+	-	yes	yes
16	21	F	14.8	**<0.05**	0.73	558	482	1724	1103	494	-	L171R	+	-	-	yes	n.t.
17	28	M	9.8	**<0.05**	1.57	**85**	**59**	2063	986	1012	-	-	+	-	-	**no**	yes
18	28	F	14.3	**<0.05**	1.15	139	100	1398	908	412	-	-	+	-	psoriasis, psoriatic arthritis	yes	n.t.
19	32	F	**19.9**	**<0.04**	0.68	n.t.	n.t.	n.t.	n.t.	n.t.	n.t.	-	+	+	-	no	n.t.
20	35	F	15.3	**0.05**	0.97	244	173	1922	809	765	-	-	+	-	-	n.t.	n.t.
21	32	F	15.8	**0.05**	2.14	450	188	1935	1206	598	n.t.	-	+	-	-	n.t.	yes
22	38	M	**21.3**	**0.07**	0.47	478	397	1597	851	587	+	-	+	-	psoriasis	yes	yes
23	39	F	17	**<0.05**	1.89	240	**80**	2090	1199	746	+/-	-	+	NA	Graves' disease	**no**	**no**
24	39	M	16.3	**0.05**	1.22	169	128	621	325	235	n.t.	n.t	+	+	-	n.t.	n.t.
25	51	F	**20.1**	**0.05**	1.99	416	229	1465	808	558	+/-	A181E	+	-	-	yes	n.t.
26	59	F	13.5	**<0.05**	0.39	**86**	130	1261	797	443	n.t.	-	+	-	-	n.t.	n.t.
27	61	F	**17.4**	**<0.06**	0.73	n.t.	n.t.	n.t.	n.t.	n.t.	n.t.	n.t.	-	NA	NA	n.t.	n.t.

28	66	F	16.7	**<0.01**	1.34	162	426	1275	622	585	n.t.	-	+	NA	psoriasis	yes	yes
29	74	F	**21.6**	**<0.07**	1.29	n.t.	n.t.	n.t.	n.t.	n.t.	n.t.	n.t.	+	NA	NA	n.t.	n.t.
30	84	M	10.3	**<0.06**	**3.11**	n.t.	n.t.	n.t.	n.t.	n.t.	n.t.	n.t.	+	NA	NA	n.t.	n.t.
Normal range	3-5 yrs		5- 13	0.5- 2.3	0.3-1.8	200-2100	100-1000	900-4500	500-2400	300- 1600							
	6- 9 yrs		6- 13	0.5- 3.0	0.4-1.6	200-1600	90- 600	700-4200	300-2000	300- 1800							
	10-16 yrs		7- 15	0.5-4.0	0.4-1.8	200-600	70- 1200	800-3500	400-2100	200- 1200							
	16- 18 yrs			0.7- 4.0	0.4-2.3	100-500	90- 600	700-2100	300-1400	200- 900							
	> 18 yrs		7- 16	0.8- 3.9	0.45-2.3	100-500	90- 600	700-2100	300-1400	200- 900							

Subnormal values are given in bold font; supranormal underlined; yrs, years; n.t., not tested; +/-, borderline positive; NA, not available; Pneumovax, *Pneumococcus* polysaccharide vaccine; HiB, *Haemophilus influenza* type B polysaccharide-protein conjugate vaccine

4

Supplementary Table 2. Antibodies used for flow cytometric immunophenotyping.

Antibody	Clone	Fluorochrome	Supplier
CD3	UCHT1	BV711	BD Biosciences, San Jose, CA
CD3	SK7	BV786	BD Biosciences
CD3	SK7	PE-Cy7	BD Biosciences
CD3	UCHT1	APC	BD Biosciences
CD4	OKT4	BV510	BioLegend, San Diego, CA
CD4	RPA-T4	Pacific Blue	BD Biosciences
CD8	SK1	APCH7	BD Biosciences
CD8	OKT8	Pacific Blue	BD Biosciences
CD8	RPA-T8	PE-Cy5	BD Biosciences
CD14	M5E2	Pacific Blue	BD Biosciences
CD19	J3-119	PC7	Beckman Coulter, Fullerton, CA
CD16	NKP15	PE	BD Biosciences
CD20	2H7	BV510	BioLegend
CD21	B-ly4	BV711	BD Biosciences
CD21	B-Ly4	PE	BD Biosciences
CD23	EBVCS-5	APC	BD Biosciences
CD24	ML5	FITC	BD Biosciences
CD25	BC96	BV421	BioLegend
CD25	2A3	PE	BD Biosciences
CD27	O323	BV421	BioLegend
CD27	M-T271	FITC	BD Biosciences
CD28	CD28.2	PerCP-Cy5.5	BioLegend
CD38	HIT2	BV605	BioLegend
CD38	HIT2	APC	BD Biosciences
CD45	HI30	FITC	BD Biosciences
CD45	2D1	V450	BD Biosciences
CD45RA	HI100	FITC	BD Biosciences
CD45RA	HI100	BV605	BioLegend
CD45RO	UCHL1	FITC	DAKO, Glostrup, Denmark
CD56	B159	PE	BD Biosciences
CD69	FN50	BV711	BD Biosciences
CD79B	SN8	PerCP-Cy5.5	BD Biosciences
CD80	2D10	BV605	BioLegend
CD86	IT2.2	PE-Cy7	BioLegend
CD95	DX2	FITC	BD Biosciences
CD127	A019D5	APC	BioLegend
CCR4	TG6/CCR4	PECy7	BioLegend
CCR6	G034E3	PerCP-Cy5.5	BioLegend
CCR7	REA108	PE	Miltenyi Biotec, Cologne, Germany
CCR7	3D12	PE	BD Biosciences
CCR10	6588-5	PE	R&D systems, Minneapolis, MN
CXCR3	G025H7	FITC	BioLegend
CXCR5	51505	APC	R&D systems
IgA	IS11-8E10	FITC	Miltenyi Biotec
IgA	IS11-8E10	APC	Miltenyi Biotec
IgD	IA6-2	PECF594	BD Biosciences

IgD	IA6-2	PE	BD Biosciences
IgG	G18-145	PE	BD Biosciences
IgM	MHM-88	BV510	BioLegend
IgM	G20-127	PE-Cy5	BD Biosciences
TACI	1A1	APC	BD Biosciences
TACI	1A1	PE-DAZZLE	BioLegend
TCRγδ	11F2	PECy7	BD Biosciences
Viability dye		AF700	BD Biosciences

Supplementary Table 3. Primer sequences for PCR and sequencing of the *TNFRSF13B* gene.

Exon	Forward primer (5’- 3’)	Reverse primer (5’- 3’)
1	GCCCGGCAGGCCTTCCACT	GCAAGCCCCACATCCCAGAGG
2	GCCCCAGGCCCCTAGCAAGTG	TCTCCTCCTGCCACCCTTTCCTCA
3	GGCTTACTCTGGAATTGCCTTCTG	CTTCTGGCCATTTGCTTGGACT
4	CCAGCCTCTCCAGGAGCCAGAC	CCGGGTGCCACTCTCCCAGTTA
5	CCTGGGTCGGGGGAGAGTG	TGCCCCGACCTCCTGCTCTATC

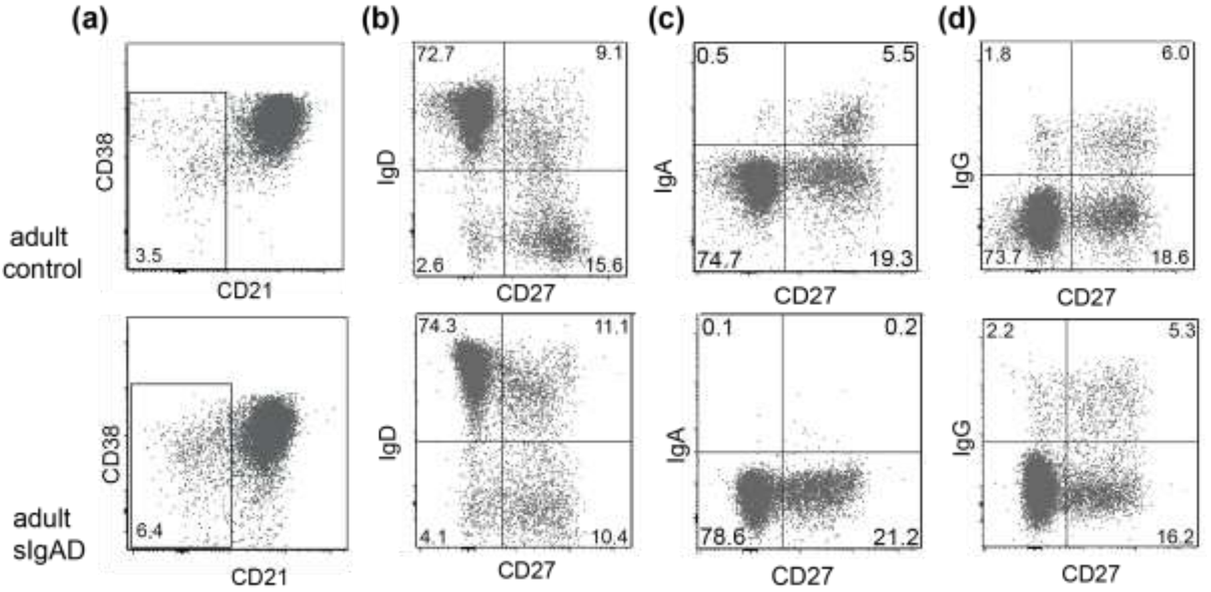

Supplementary Figure 1. Gating strategy B-cell subsets. Representative dot plots of CD19+ B cells showing gating strategy for **(a)** CD21^{low} B cells, **(b)** naive B cells (CD27^{-}IgD^{+}), IgD+CD27+ memory B cells, **(c)** IgA memory B cells and **(d)** IgG memory B cells.

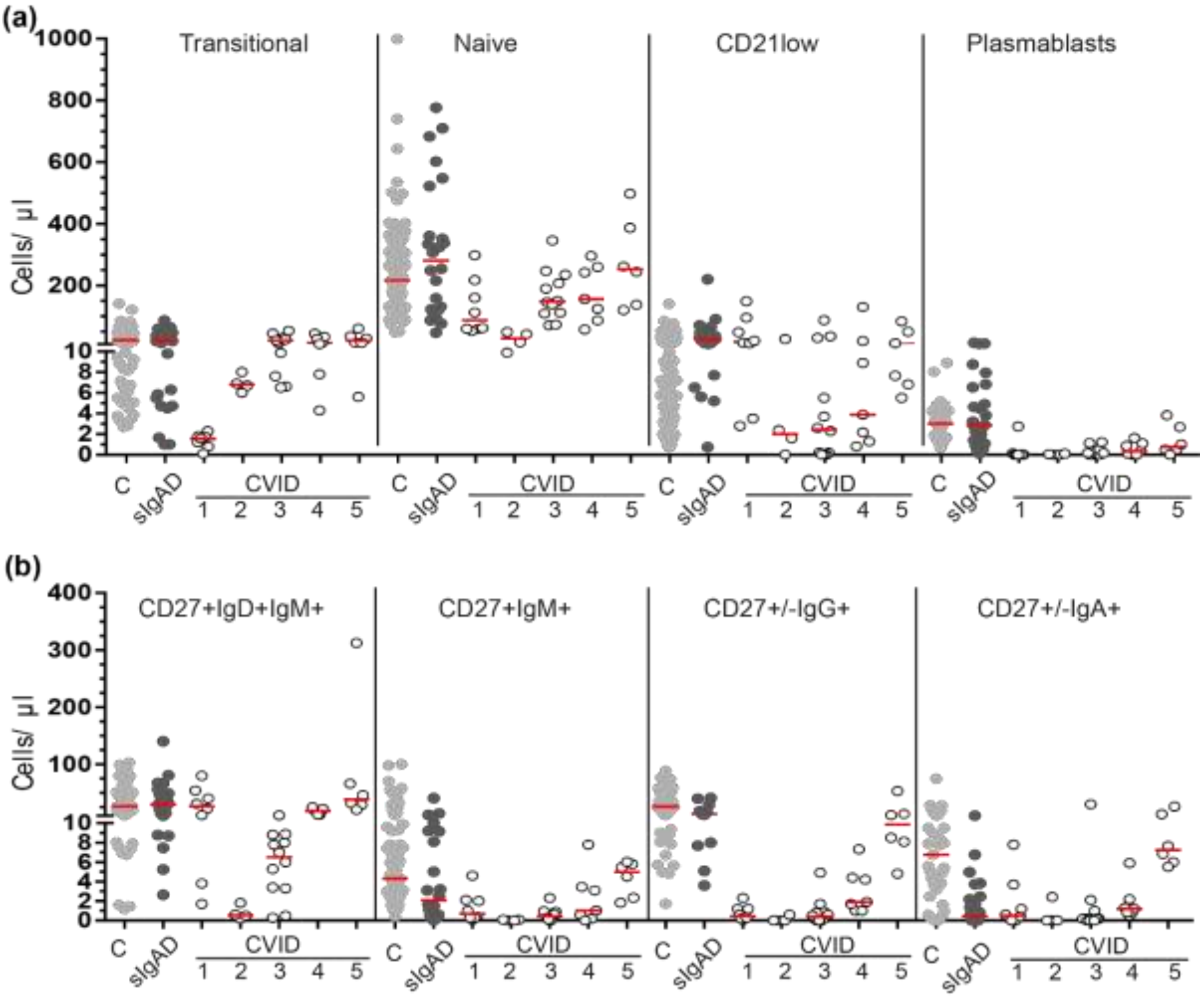

Supplementary Figure 2. B-cell subsets of sIgAD patients and CVID patients. **(a)** Absolute numbers of total transitional, naive, CD21^{low} B cells and plasmablasts. **(b)** Absolute numbers of memory B-cell subsets: CD27^{+}IgD^{+}IgM^{+}, CD27^{+}IgM^{+}, IgG^{+} memory (CD27^{-} and CD27^{+}) and IgA^{+} memory (CD27^{+} and CD27^{-}) B cells. Each dot represents one sample; B-cell pattern in 37 CVID patients: 1 (n=8), 2 (n=4), 3 (n=12), 4 (n=7), 5 (n=6). Controls (C) n=101, sIgAD patients n=26 (except for CD27^{+}/$^{-}$IgG^{+}, n=11). Red lines represent median values.

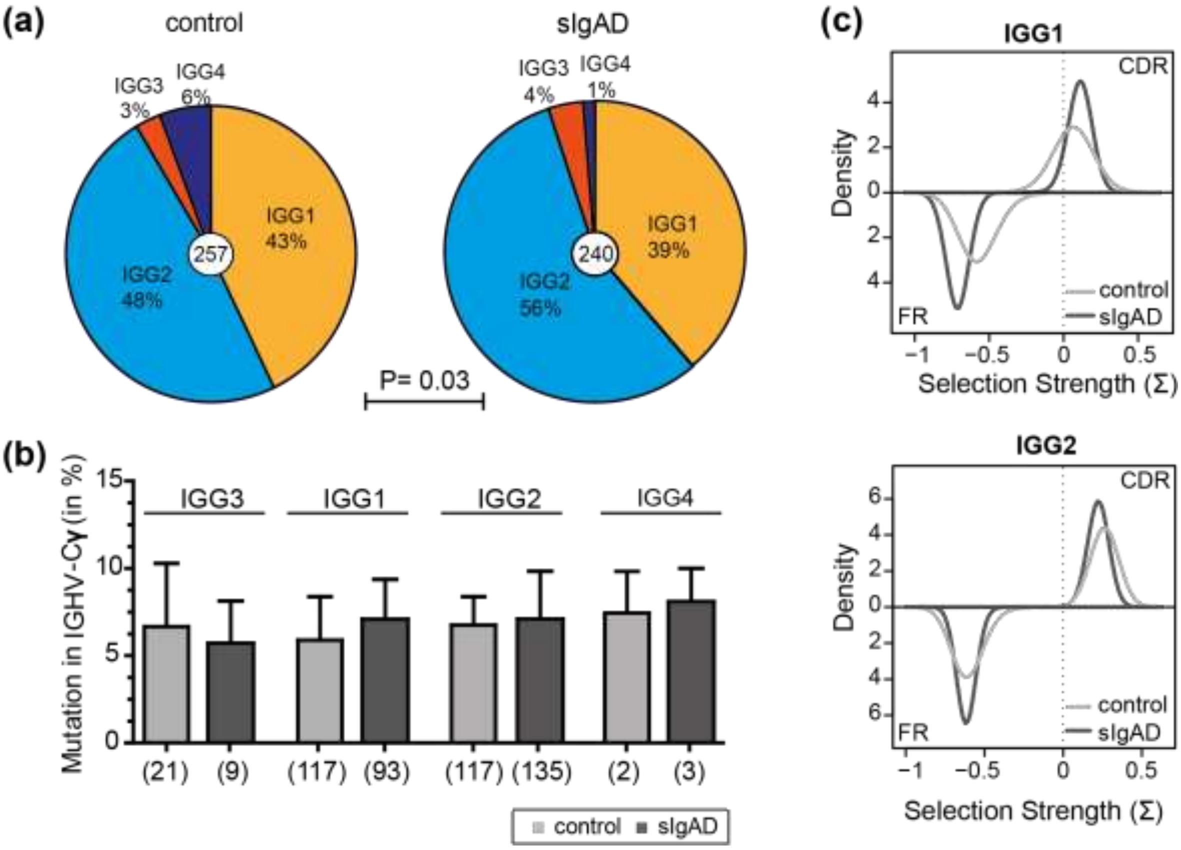

Supplementary Figure 3. IgG class switch recombination and somatic hypermutation in adult sIgAD patients. **(a)** IgG subclass distribution in unique transcripts with the number of analyzed sequences depicted in the white circle. **(b)** Frequencies of SHM in *IGG* transcripts per subclass; numbers of unique sequences are indicated in parenthesis. **(c)** Antigenic selection of SHM in *IGG1* and *IGG2* subclass transcripts. *IGG3* and *IGG4* transcripts were not included in this analysis due to low numbers. Statistics calculated with Mann-Whitney *U* test.*, $P < 0.05$.

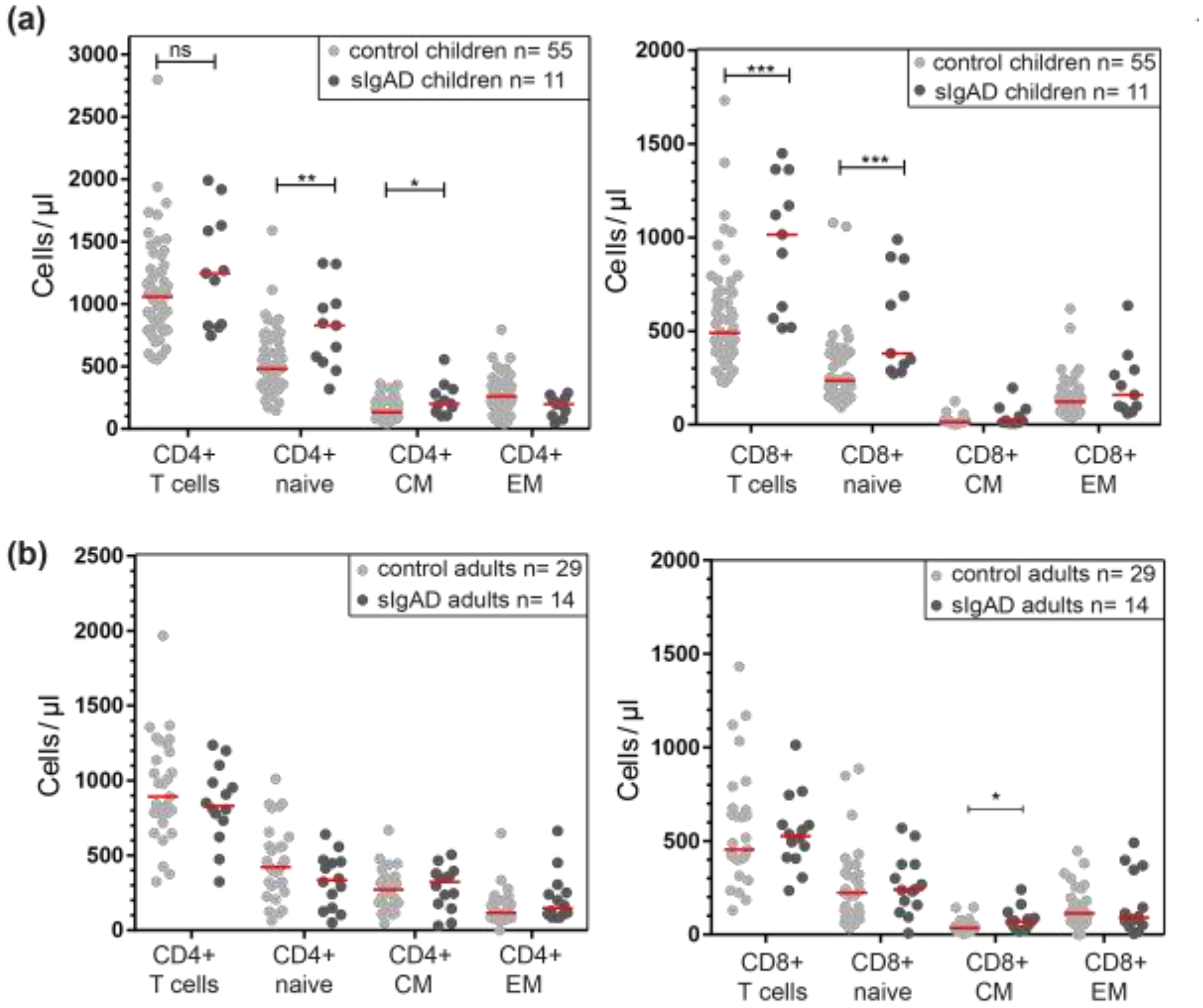

Supplementary Figure 4. Naive and memory CD8 and CD4 T-cell subsets in sIgAD patients. **(a)** Absolute numbers of total CD4$^+$ T cells, CD4$^+$ naive T cells (CD45RO$^-$CCR7$^+$), CD4$^+$ central memory (CM) T-cells (CD45RO$^+$CCR7$^+$), CD4$^+$ effector memory (EM) T cells (CD45RO$^{+/-}$CCR7$^-$), total CD8$^+$ T cells, CD8$^+$ naive T cells (CD45RO$^-$CCR7$^+$), CD8$^+$ central memory (CM) T-cells (CD45RO$^+$CCR7$^+$) and CD8$^+$ effector memory (EM) T cells (CD45RO$^{+/-}$CCR7$^-$) in children. **(b)** Absolute numbers of T-cell subsets in adults, as defined as in panel **(a)**. Each dot represents one subject; red lines indicate median values. Statistics calculated with the Mann-Whitney *U* test; *** $P < 0.001$; ** $P < 0.01$; * $P < 0.05$.
